# The Metric System

**Published:** 1878-07

**Authors:** J. B. Hodgkin


### ARTICLE III.

### *The Metric System.*

By order of the Surgeon General at Washington, the "Metric System" is ordered to be used in all Marine Hospital Service of the United States. A small pamphlet has been published for the guidance of those concerned, and from it we compile such facts relating to this system as may seem to be of interest to the general reader. As the "Metre," "Centimetre," &c., are now in somewhat common use, it is well to have before us in shape convenient for reference, a synopsis of the system.

### RULES.

---

1. To Express Quantities by Weight of the Apothecaries' System in Metric Terms, or to Write Medical Prescriptions in Metric Weights.

Rule A.—*Reduce each quantity to grains; then divide the number by* 10, (or move the decimal point one place to the left,) *and from the quotient subtract one-third.* The remainder is in each case the number of grammes representing (nearly) the same quantity. Or,

Rule B.—*Reduce each quantity to drachms, and multiply the number by* 4. The product is in each case the number of grammes representing (nearly) the same quantity, Or,

Rule C.—*Reduce each quantity to ounces, and multiply the number by* 32. The product is in each case the number of grammes representing (nearly) the same quantity.

One gramme is equal to 15.43234874 troy grains.—(*Prof. Miller.*) In preparing the above rules the fraction has been ignored, as for medical and pharmacal purposes 1 gramme and 15 troy grains may be safely considered as equal quantities. In rule A, therefore, a division by 15 may, if preferred, be substituted for the division by 10 followed by a subtraction of one-third from the quotient, with the same result. The difference between 15 and 15.43234874 is 2.882 + per cent., and hence the deviation from exactness in the answer arrived at by either of the above rules corresponds to an excess of 28.82 + grains for every 1,000 grains. To illustrate: By Rule B, 4,000 grammes would be (nearly) equivalent to 1,000 drachms; but 4,000 grammes is equal to exactly 61,729.40 + troy grains, while 1,000 drachms is only 60,000 troy grains. The deviation from exactness, therefore, in the answer arrived at by Rule B, (as also in the answers arrived at by Rules A and C,) is equivalent to an excess of 1,729.40 + troy grains for every 1,000 drachms, or about 14 grains for every ounce, or 28.82 + grains for every 1,000 grains, or less than 2.9 per cent.

To insure greater accuracy, if in any case deemed necessary, three per cent. may be deducted from the answer arrived at by either of the Rules A, B, or C. The deviation from exactness will then be reduced to one-fifth of one per cent., the remainder being less than the exact equivalent sought by only 2.04 grains for every 1,000 grains, or about one grain for every ounce.

2. To Express Quantities by Measure of the Apothecaries' System in Metric Terms, or to Write Medical Prescriptions in Metric Cubic Measures.

Rule D.—*Reduce each quantity to minims; then divide the number by* 10, (or move the decimal point one place to the left,) *and from the quotient subtract one-third.* The remainder is in each case the number of cubic centimetres representing (nearly) the same quantity. Or,

Rule E.—*Reduce each quantity to fluid drachms, and multiply the number by* 4. The product is in each case the number of cubic centimetres representing (nearly) the same quantity. Or,

Rule F.—*Reduce each quantity to fluid ounces, and multiply the number by* 32. The product is in each case the number of cubic centimetres representing (nearly) the same quantity.

One metre is equal to 39.370432 inches.—(*Capt. Clarke.*) Hence one cubic centimetre is equal 0.0610253868 — cubic inches, or to 16.2311678 + minims, (there being 61,440 minims in each wine-gallon of 231 cubic inches.) In preparing the above rules 1 cubic centimetre and 15 minims have been considered as equal quantities, which, for medical and pharmacal purposes, is deemed sufficiently accurate. In Rule D, therefore, a division by 15 may, if preferred, be substituted for the division by 10 followed by a subtraction of one-third from the quotient, with the same result. The difference between 15 and 16.2311678 + is 8.208 — per cent., and hence the deviation from exactness in the answer arrived at by either of the above rules corresponds to an excess of 82.08— minims for every 1,000 minims. To illustrate: By Rule E, 4,000 cubic centimetres would be (nearly) equivalent to 1,000 fluid drachms; but 4,000 cubic centimetres is equal to exactly 64,924.67+ minims, while 1,000 fluid drachms is only 60,000 minims. The deviation from exactness, therefore, in the answer arrived at by Rule E, (as also in the answers arrived at by Rules D and F,) is equivalent to an excess of 4,924.67+ minims for every 1,000 fluid drachms, or about 41 minims for every fluid ounce, or 82.08— minims for every 1,000 minims, or 8.2 per cent.

To insure greater accuracy, if in any case deemed necessary, 8 per cent. may be deducted from the answer arrived at by either of the Rules D, E, or F. The deviation from exactness will then be reduced to less than one-half of one per cent., the remainder being less than the exact equivalent sought by only 4.49— minims for every 1,000 minims, or less than $2\frac{1}{2}$ minims for every fluid ounce.

The important advantage of a simple relation between the units of weight and the units of measure is acknowledged, and is one of the strong arguments in favor of the metric system, the weight unit or "gramme" being the weight of one cubic centimetre of distilled water of maximum density.

under the pressure of one atmosphere. The minim and the grain, however, have no simple relation to each other; but as the difference between the weight of one minim of distilled water of maximum density under the pressure of one atmosphere, and the weight of a troy grain, is comparatively small, it has been ignored entirely in preparing the rules for the conversion of apothecaries' measure into metric measure, (Rules D, E, and F,) and hence the arithmetical processes in the rules for converting old measures into new are respectively identical with the processes given in the rules for converting weights, as will be seen upon comparison of Rule D with Rule A, Rule E with Rule B, and Rule F with Rule C. For this purpose one minim is considered as weighing one grain. one fluid drachm as weighing one drachm, and one fluid ounce as weighing one ounce.

It will be seen that if the three Rules A, B, and C, be all applied in converting the several quantities by weight in any one prescription or formula, *the original proportions between these quantities will still be preserved*, the deviation from exactness being invariable. It will also be seen that the three Rules D, E, and F, may be all applied in converting the several quantities by measure in any one prescription or formula *without disturbing the original proportions* between said quantities. Thus, if all the ingredients in the formula be expressed by weight, or if they all be expressed by weight, or if they all be expressed by measure, the rules given may be employed indiscriminately without changing the character of the formula in the least. But if in any one formula both weights and measures are used together, then the proportions between the quantities by weight and the quantities by measure will be changed, so that, in the metric formula, constructed according to the rules given, the measured quantities will be about five per cent. larger in proportion to the weighed quantities, the deviation from exactness in the measures being an excess of eight per cent., while in the weights it is an excess of only three per cent. Thus, if a prescription for one grain of a strychnia salt dissolved in four fluid ounces of water, be converted into metric terms by the application of these rules, the metric formula arrived at would give us a solution *five per cent. weaker*, which is an absolutely insignificant difference.

In applying the foregoing rules for writing prescriptions, the metric quantities should be adjusted so as to be expressed in as simple decimal terms as may be practicable, without materially changing the dose or the character of the formula.

The terms "gramme" and "cubic centimetre" might be abbreviated "*Gm.*" and "C. C." To preclude the possibility, (in *careless* writing,) however, of mistaking the sign "*Gm.*" (gramme) for the sign "gr.," (grain,) the number should invariably precede the sign, using the common Arabic numerals. Thus, while ten grains is always written "gr. X," (Roman numerals being used,) ten grammes would be written "10 *Gm.*" When the term "centigramme" is used it should be spelled out in full. Ten centigrammes might, however, more conveniently be written "0.10 *Gm.*" than "10 centigrammes." In writing, the abbreviated metric denominations should always be underscored; but the preceding number should not.

Two examples will suffice to illustrate the foregoing rules and suggestions. The following prescription—

℞: Extr. Coloc. Comp., ʒ iss. Extr. Colch. Acet., gr. xii. Extr. Digitalis, gr. vj. Make into 24 pills—

would in metric terms, be written:

℞: Extr. Coloc. Comp., 6 *Gm.* (See Rule B.) Extr. Colch. Acet., 0.8 *Gm.* (See Rule A.) Extr. Digitalis, 0.4 *Gm.* (See Rule A.) Make into 24 pills.

Or, in a more finished decimal manner:

℞: Extr. Coloc. Comp., 7.50 *Gm.* Extr. Colch. Acet., 1 *Gm.* Extr. Digitalis, 0.5 *Gm.* Make into 30 pills.

And the following prescription—

℞: Potassii Bromidi, ℥ i. Elix. Aurantii, fl. ℥ viij. Mix.

would, in metric terms, be written:

℞: Potassii Bromidi, 32 *Gm.* (See Rule C.) Elix. Aurantii, 256 C. C. (See Rule F.) Mix.

Or, in a more finished decimal manner:

℞ : Potassii Bromidi, 30 *Gm.* Elix. Aurantii, 250 C. C. Mix.

The exact equivalents of the grain, drachm, and ounce, (troy,) in grammes: of the gramme in grains; of the minim, fluid drachm, and fluid ounce in cubic centimetres, and of the cubic centimetre in minims, are as follows:

1 grain, troy, is equal to 0.065— grammes. 1 drachm, troy, is equal to 3.888— grammes. 1 ounce, troy, is equal to 31.103+ grammes. 1 gramme is equal to 15.43234874 grains, troy.—*Prof. Miller.*

(1 avoirdupois pound is equal to 453.592+ grammes.) (1 avoirdupois ounce is equal to 28.350+ grammes.) 1 minim is equal to 0.062— cubic centimetres. 1 fluid drachm is equal to 3.697— cubic centimetres. 1 fluid ounce is equal to 29.573—cubic centimetres. 1 cubic centimetre is equal to 16.231+ minims. (1 metre is equal to 39.370432 inches.—*Capt. Clarke.*) H.

